# Adsorption of SF_6_ decomposed gas on anatase (101) and (001) surfaces with oxygen defect: A density functional theory study

**DOI:** 10.1038/srep04762

**Published:** 2014-04-23

**Authors:** Xiaoxing Zhang, Qinchuan Chen, Ju Tang, Weihua Hu, Jinbin Zhang

**Affiliations:** 1 State Key Laboratory of Power Transmission Equipment & System Security and New Technology, Chongqing University, Chongqing 400044, China; 2 School of Electrical Engineering, Wuhan University, Wuhan 430072, China; 3 Institute for Clean Energy & Advanced Materials, Southwest University, Chongqing 400715, China; 4 Chongqing Power Company, Beibei, Chongqing 400700, China

## Abstract

The detection of partial discharge by analyzing the components of SF_6_ gas in gas-insulated switchgears is important to the diagnosis and assessment of the operational state of power equipment. A gas sensor based on anatase TiO_2_ is used to detect decomposed gases in SF_6_. In this paper, first-principle density functional theory calculations are adopted to analyze the adsorption of SO_2_, SOF_2_, and SO_2_F_2_, the primary decomposition by-products of SF_6_ under partial discharge, on anatase (101) and (001) surfaces. Simulation results show that the perfect anatase (001) surface has a stronger interaction with the three gases than that of anatase (101), and both surfaces are more sensitive and selective to SO_2_ than to SOF_2_ and SO_2_F_2_. The selection of a defect surface to SO_2_, SOF_2_, and SO_2_F_2_ differs from that of a perfect surface. This theoretical result is corroborated by the sensing experiment using a TiO_2_ nanotube array (TNTA) gas sensor. The calculated values are analyzed to explain the results of the Pt-doped TNTA gas sensor sensing experiment. The results imply that the deposited Pt nanoparticles on the surface increase the active sites of the surface and the gas molecules may decompose upon adsorption on the active sites.

Sulfur hexafluoride (SF_6_) is widely used in gas insulated switchgear (GIS) because of its excellent insulating and arc-extinguishing abilities. At the early stages of the electrical equipment insulation degradation, partial discharge (PD) easily occurs and causes the decomposition of SF_6_ into various products^1^. The composition and content of these products can be used to determine the types, sizes, and causes of the partial discharge and provide reliable information to evaluate the insulation status of the equipment^2^,^3^,^4^,^5^. Gas sensors can be used to detect the composition and content of mixed gases^6^. A TiO_2_ nanotube array (TNTA) gas sensor is used in this study to detect characteristic by-products of SF_6_, such as SO_2_, SOF_2_, and SO_2_F_2_.

TNTA is a typical 3D nanomaterial that has a large specific surface area and a nanometer size effect^7^. The gas molecules can easily adsorb on the surface of TNTAs and affect the conductive properties^8^. The TNTA sensor possesses advantages such as fast response, low detection limit, high sensitivity, and good stability. TNTA gas sensors that have been studied include common gases such as H_2_, O_2_, NH_3_, and NO_2_^9^. However, detecting the decomposition by-products of SF_6_ using the TNTA gas sensor has been seldom reported. Here, we prepared intrinsic and Pt-doped TNTAs. The preliminary gas-sensing experiment results indicate that TNTAs can be used to detect certain characteristic gases under different concentrations^10^,^11^.

The TNTAs prepared are mainly anatase, whose surface is dominated by thermodynamically stable (101) facets and has few oxygen vacancy defects. Based on the gas-sensing experiment, the first-principles density functional theory (DFT) calculations are adopted to analyze the adsorption of SO_2_, SOF_2_ and SO_2_F_2_ on the anatase (101) perfect surface^12^. However, some researches show that the oxygen vacancy defects can form on the surface of TNTAs, and depend on the oxygen content of the annealing environment^13^,^14^. Low oxygen content is low easily forms the oxygen vacancy on the surface. In addition, this reference shows that the anatase, whose surfaces are mainly activity (001) facets, can be prepared by the hydrothermal method^15^,^17^. Compared with the (101) surface, the (001) surface of anatase has higher activity but has lower thermal stability.

In this paper, the adsorption behaviors of these gases on anatase (101) surface and (001) surface are studied in new models using the first-principles DFT calculations to further explore the TNTA sensing on SO_2_, SOF_2_, and SO_2_F_2_. Given the possible occurrence of an oxygen vacancy, the surfaces include (101) perfect surface, (101) defect surface, (001) perfect surface, and (001) defect surface. We analyzed the response characteristic of Pt-doped TNTAs to the three gases based on the simulation results, and provide a theoretical rationale for TNTAs applied in detecting the decomposition by-products of SF_6_.

## Computational details

We used a periodic boundary model, and the sizes of the (101) and (001) surface supercell are 10.88 Å × 7.55 Å × 17.84 Å and 7.62 Å × 7.62 Å × 17.84 Å. The height of vacuum above the surface is about 12 Å to avoid the interaction between the adjacent cells which are induced by the periodic boundary condition. The generalized gradient approximation (GGA) exchange-correlation function parameterized by Perdew et al. is employed for the electron-electron exchange and the correlation interactions^18^,^19^. The double numerical basis set plus polarization functions (DNP) are used^20^,^21^. All optimized structures are obtained with a precision of 1 × 10^−5^ Ha for the energy, 2 × 10^−3^ Ha/Å for the force, and 5 × 10^−3^ Å for the displacement. The convergence threshold for the electronic self-consistent field is 1 × 10^−6^ Ha, and a Fermi smearing of 5 × 10^−4^ Ha is used. To hasten the convergence and reduce calculation time, the direct inversion in an iterative subspace (DIIS) is used simultaneously. The Brillouin zones of the (101) and (001) surface models are respectively sampled at 3 × 1 × 2 and 3 × 3 × 1 *k*-meshes^22^. All spin-unrestricted DFT calculations are performed using the Dmol^3^ package.

**Figure 1:**
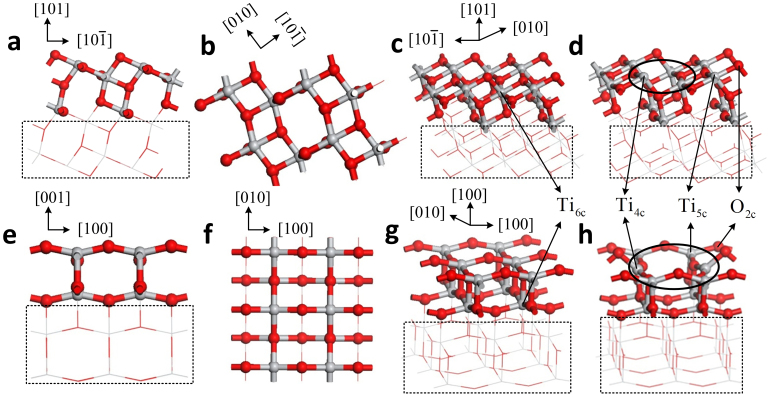
Four kinds of surfaces. (a), (b) and (c) are the views of (101) perfect surface. (d) (101) defect surface. (e), (f) and (g) are the views of (001) perfect surface. (h) (001) defect surface. Ti and O atoms are shown in gray and red, respectively. Ti_6c_, Ti_5c_ and O_2c_ are marked by arrows and oxygen vacancy sites are marked by ellipse.

**Figure 2:**
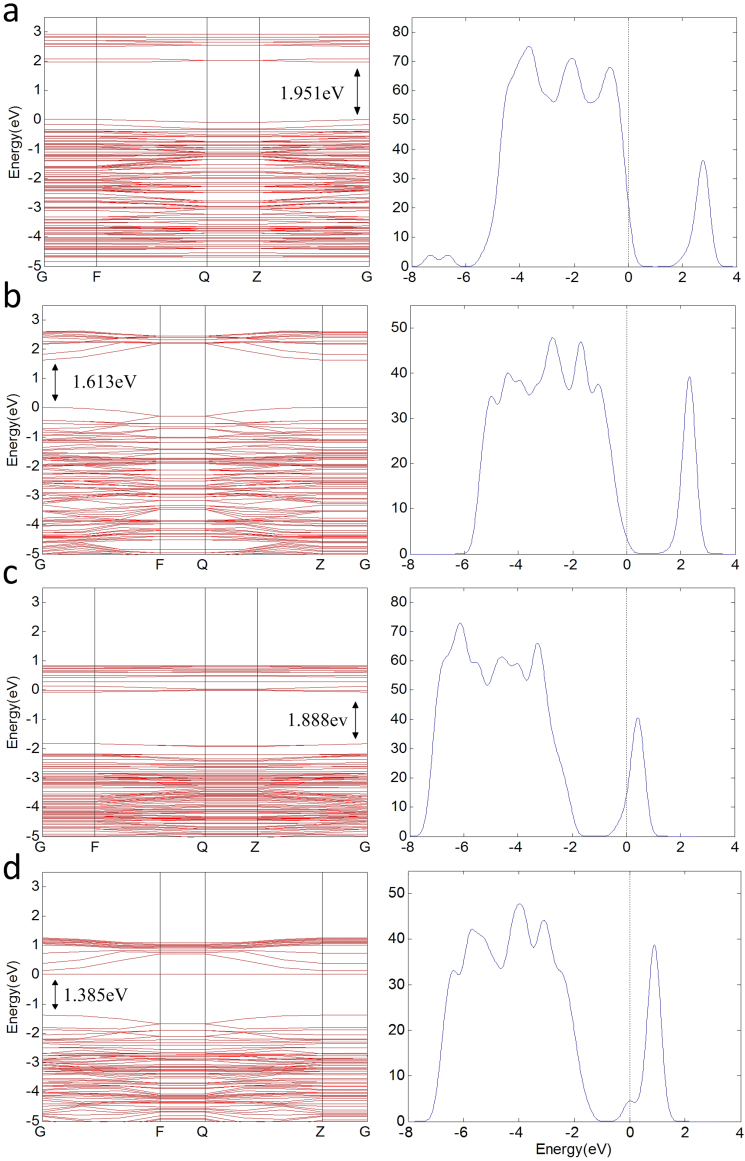
Band structures and density of states. (a) TDOS of (101) perfect surface and its band gap is 1.951 eV. (b) The TDOS of (001) perfect surface and its bang gap is 1.612 eV. (c) TDOS of (101) perfect surface and its band gap is 1.888 eV. (d) TDOS of (001) defect surface and its band gap is 1.385 eV. The Fermi level is aligned at zero.

**Figure 3:**
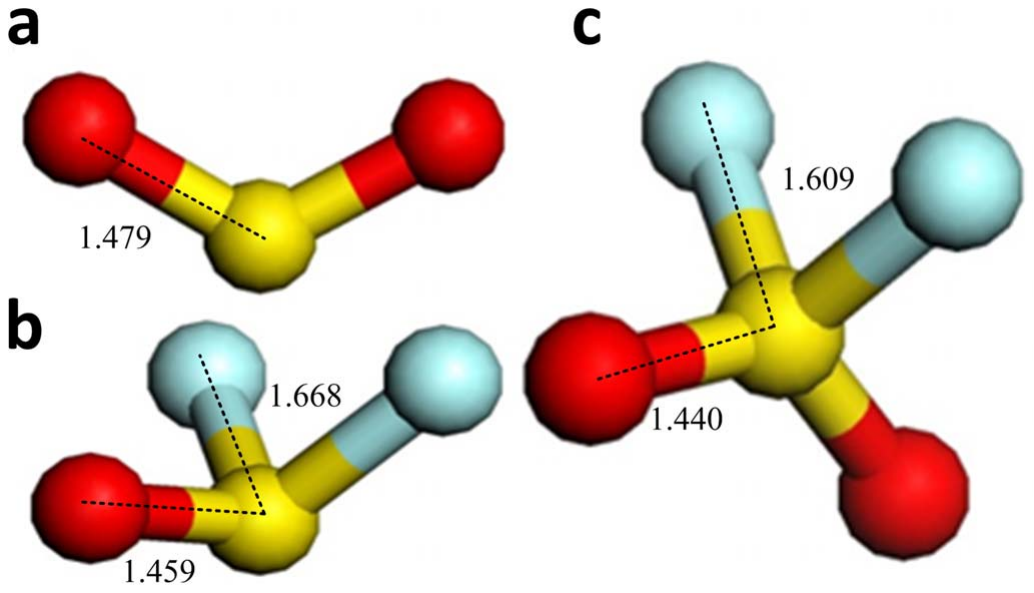
Structure of the gas molecules. (a) SO_2_ molecule and its S-O bond is 1.479Å. (b) SOF_2_ molecule, its S-O bond is 1.459Å and its S-F bond is 1.668Å. (c) SO_2_F_2_ molecule, its S-O bond is 1.440 Å and its S-F bond is 1.609Å. S, O, and F atoms are respectively shown in red, yellow, and blue.

Figures 1 and 2 illustrate the four kinds of surfaces, as well as their band structures and density of states. Defect levels at the surface facilitate conduction along it, but the band gap of the bulk anatase does not change. Figure 3 shows the molecule models of the three gases. Optimizing the surface structures fixes the atoms in the lower part in the structure and only optimizes the atoms in the upper part. Upon optimizing the adsorption structures, the three gas molecules should approach the active atoms on the surface in different orientations and behavior to determine the most stable adsorption structures.

## Results and discussion

The gas–surface interaction can be described using the adsorption energy *E*_ad_ defined as *E*_ad_ = *E*_surf/gas_− *E*_gas_− *E*_surf_, where *E*_gas_ is the energy of one isolated gas molecule in its optimized structure, *E*_surf_ is the total energy of the TiO_2_ structure, and *E*_surf/gas_ is the total energy of the adsorption structure. *E*_ad_<0 implies that the adsorption process is exothermic and spontaneous.

The change in charge distribution should be known. Therefore, the charge transfer *q* is calculated using the Mulliken population analysis defined as the charge variation of isolated gas molecules upon adsorption. *q* > 0 implies that the charge is transferred from the molecule to the anatase surface. The binding distance *d* is defined as the nearest distance between the molecule and the surface. The energy gap of adsorption structure can be calculated through the energy levels of the HOMO and LUMO defined as *E*_g_ = |*E*_HOMO_ − *E*_LUMO_|. The calculated adsorption parameters of the three gases on the four kinds of anatase surfaces are shown in table 1, table 2 and table3.

### SO_2_, SOF_2_, and SO_2_F_2_ gas molecule adsorption on the anatase (101) and (001) perfect surface

**Figure 4:**
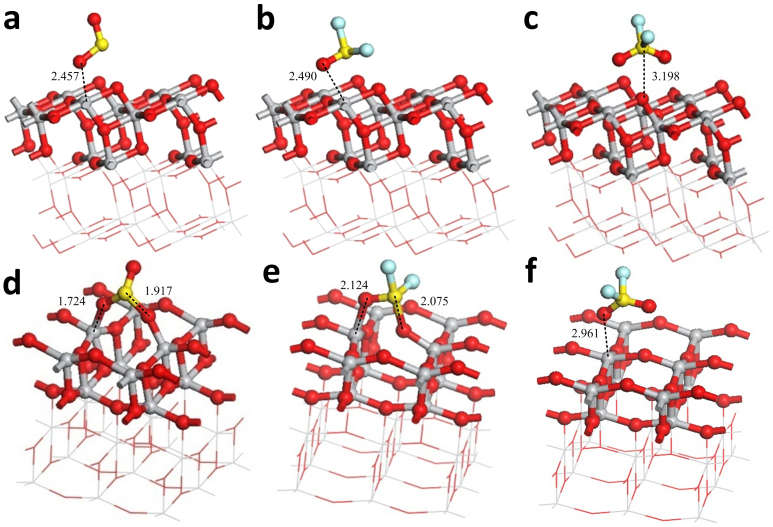
Adsorption structures of gas molecules on perfect surface. (a) SO_2_ adsorbs on the (101) perfect surface. (b) SOF_2_ adsorbs on the (101) perfect surface. (c) SO_2_F_2_ adsorbs on (101) perfect surface. (d) SO_2_ adsorbs on the (001) perfect surface. (e) SOF_2_ adsorbs on the (001) perfect surface. (f) SO_2_F_2_ adsorbs on the (001) perfect surface. Binding distances are in Å.

Figures 4(a) to 4(c) show the most stable adsorption structure of the three gas molecules on the (101) perfect surface. In table 1, the energies released in the adsorptions and the charge transfers are small and follow the order SO_2_ > SOF_2_ > SO_2_F_2_. This result reveals that the three gas molecules are physisorbed (not chemisorbed) on the (101) perfect surface and coincides with the results supplied by the Ref. 11.

Figures 4(d) to 4(f) illustrate the most stable adsorption structure of the three gas molecules on the (001) perfect surface. Similar to the adsorption structures of (101) surface, the energies released during adsorption and the charge transfers are all greater than that on (101) perfect surface, but follow the order SO_2_ > SOF_2_ > SO_2_F_2_. This result implies that the three gas molecules have stronger interaction with the (001) surface. The energy of SO_2_ adsorbed on the (001) surface is 1.66 eV, which is greater than 0.6 eV, and suggests that SO_2_ is chemisorbed on the (001) surface. The adsorption of SO_2_ and SOF_2_ on the (001) surface strengthens the interactions between the molecules and the surface, and the charge is transferred from the surface to the molecule. The adsorptions of SO_2_F_2_ on the (001) surface and the three gas molecules on the (101) surface weaken the interactions and the charge is transferred from the molecule to the surface. A strong interaction transfers the charge from the molecule to the surface.

Comparison of the (101) and (001) surfaces reveals that the (001) surface is more active than the (101) surface, because the Ti-O bond of the former is longer than that of the (101) surface. Ti_5c_ and O_2c_ on the (001) surface is more active and easier to interact with the gas molecules. Hence, the ability of (001) surface to absorb gas molecules is better than that of (101) surface at room temperature. The gas sensor, whose surface is (101) surface, requires a high temperature to yield a high response during the gas-sensing experiment. Based on this phenomenon and the simulation results, we conclude that increasing the temperature of the (101) surface increases the length of the Ti-O bond on the surface, and the atoms become more active. Moreover, the physisorption events between the gas molecules and the (101) surface at low temperature transform to chemisorption events at high temperatures, similar to the latter between the gas molecules and the (001) surface.

**Figure 5:**
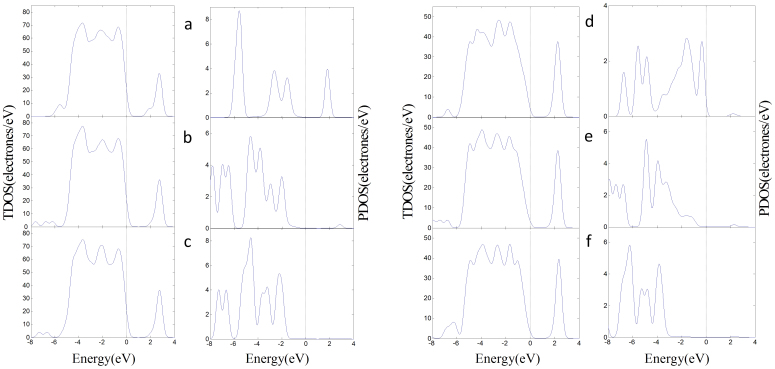
DOS of the adsorption structures on perfect surfaces. (a) The TDOS of SO_2_ adsorbs on (101) surface in left and the PDOS of SO_2_ in right. (b) SOF_2_ adsorbs on the (101) surface. (c) SO_2_F_2_ adsorbs on the (101) surface. (d) SO_2_ adsorbs on the (001) surface. (e) SOF_2_ adsorbs on the (001) surface. (f) SO_2_F_2_ adsorbs on the (001) surface.

Figure 5 illustrates the total density of states (TDOS) of the adsorption structures and the projected density of states (PDOS) of adsorbed gas molecules. The DOS reveals that the transformation of the physisorption of SO_2_ on the (101) surface to the chemisoption of SO_2_ on the (001) surface decreases the DOS of SO_2_ in CB, and that in VB is closer to the Fermi level. Here, the 2p orbitals of O in SO_2_ are hybridized with the 3d orbitals of Ti_5c_, and the 2p orbitals of S atom in SO_2_ are also hybridized with the 2p orbitals of O_2c_. SOF_2_ has the similar phenomena, whereas a little difference exists between the adsorptions of the PDOS of SO_2_F_2_ on (101) and (001) surfaces because of the low molecular activity. Combined with our previous studies, we find that the adsorption of the three gases on the surfaces reduces the bang gap of the adsorption structure and improve the conductive ability of the surfaces. SO_2_ is the best because of the lower HOMO and higher LUMO.

### SO_2_, SOF_2_, and SO_2_F_2_ gas molecule adsorption on the anatase (101) defect surface

**Figure 6:**
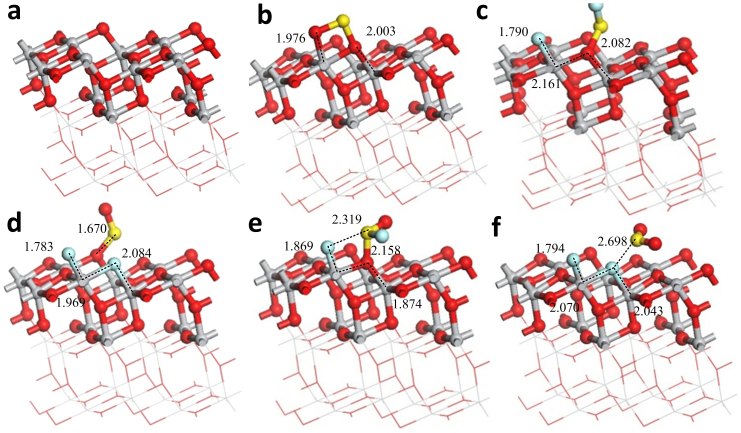
Adsorption structures of the gas molecules on the (101) defect surface. (a) The clear (101) defect surface. (b) SO_2_ adsorbs on the (101) defect surface. (c) SOF_2_ adsorbs on the (101) defect surface in the initial position F-Ti_4c_, O-Ti_5c_. (d) SOF_2_ adsorbs on the surface in F-Ti_4c_, F-Ti_5c_. (e) SO_2_F_2_ adsorbs on the surface in F-Ti_4c_, O-Ti_5c_. (f) SO_2_F_2_ adsorbs on the surface F-Ti_4c_, F-Ti_5c_. Binding distances are in Å.

Figure 6 shows the stable adsorption structure of the three gas molecules on the (101) defect surface. Different stable adsorption structures exist when the gas molecules approach the vacancies in different attitudes. Here, the oxygen vacancy defects of anatase surface are very active. The different stable adsorption structures of one gas will be discussed below.

The active atoms of the (101) defect surface are Ti_4c_ and Ti_5c_. When SO_2_ molecule adsorbs on the oxygen vacancy, one O atom of SO_2_ occupies the vacancy and the other one interacts with Ti_4c_, and Figure 6(b) shows the adsorption structure. Different stable adsorption structures exist when SOF_2_ and SO_2_F_2_ adsorb on the vacancy.

The initial positions of SOF_2_ approach the vacancy: 1) O-Ti_4c_, F-Ti_5c_, 2) F-Ti_4c_, O-Ti_5c_, 3) F-Ti_4c_, F-Ti_5c_, 4) S-Ti_4c_, O-Ti_5c_, 5) O-Ti_4c_, S-Ti_5c_. The first three have larger adsorption energies and relatively stable structures, and thus we only put the second and the third absorption structures (Figures 6(c) and 6(d)). The O atom of SOF_2_ occupies the vacancy (Figure 6(c)); and one S-F bond breaks, and the F atom interacts with Ti_4c_. Figure 6(d) shows that both of the two S-F bonds break, and one F atom occupies the vacancy; the other one interacts with Ti_4c_, whereas the SO is adsorbed on the O_2c_ of the adjacent oxygen vacancy.

The initial positions of SO_2_F_2_ approach the vacancy: 1) O-Ti_5c_, O-Ti_4c_, 2) O-Ti_4c_, F-Ti_5c_, 3) F-Ti_4c_, O-Ti_5c_, 4) F-Ti_4c_, F-Ti_5c_. The adsorption energy of first structure is small (only about 0.5 eV); the two S-F bonds do not break, but the molecular shape of SO_2_F_2_ changes greatly. The adsorption energies of the latter three stable structures have no marked differences, and thus we only put the third and the fourth absorption structures (Figures 6(e) and 6(f)). The O atom of SOF_2_ occupies the vacancy (Figure 6(e)); and one S-F bond breaks, and the F atom interacts with Ti_4c_. Figure 6(f) shows that the two S-F bonds break and the two F atoms of these bonds occupy the vacancy; the SO_2_ is absorbed on the adjacent perfect surface.

Figure 6 and Table 2 show that the interaction between the gas molecules and the oxygen vacancy of anatase surface is very strong; Because of the active Ti atoms on the surface, the S-F bonds of SOF_2_ and SO_2_F_2_ are easily broken and may produce SO_2_ and SO that will also be adsorbed on the surface.

**Figure 7:**
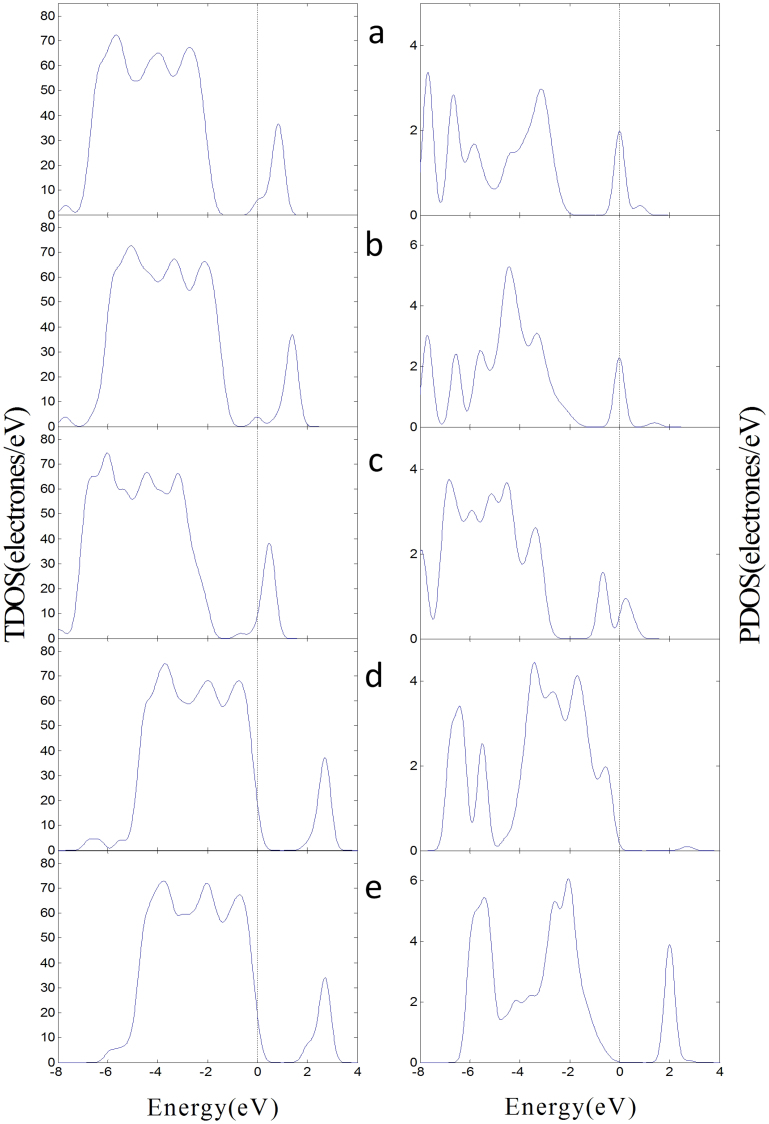
DOS of the adsorption structures on the (101) defect surface. (a) The TDOS of SO_2_ adsorbs on the (101) defect surface in left and the PDOS of SO_2_ in right. (b) SOF_2_ adsorbs on the (101) surface in the initial position F-Ti_4c_, O-Ti_5c_. (c) SOF_2_ adsorbs on the surface in F-Ti_4c_, F-Ti_5c_. (d) SO_2_F_2_ adsorbs on the surface in F-Ti_4c_, O-Ti_5c_. (e) SO_2_F_2_ adsorbs on the surface F-Ti_4c_, F-Ti_5c_.

Table 2 shows that the band gap of the (101) defect surface is 1.888 eV, which is less than that of the (101) perfect surface. This result indicates that the conductivity of the defect surface is better than that of the perfect surface. The conductivity of surface decreases when the oxygen vacancy of defect surface is occupied by O atom. The band gaps of the adsorption structures decrease and the conductivity of the (101) defect surface is improved when SO_2_ and SOF_2_ adsorb on the oxygen vacancy of (101) defect surface. Adsorption of SO_2_F_2_ increases the band gaps and reduces the conductivity of the (101) defect surface. Figure 7 illustrates the TDOS of the adsorption structures of the (101) defect surface and the PDOS of adsorbed gas molecules. Figure 7 reveals that some parts of the PDOS of SO_2_ and SOF_2_ are between the valence and conduction bands, which can improve the conductivity of the (101) defect surface. The PDOS of SO_2_F_2_ induce small changes between the VB and the CB.

### SO_2_, SOF_2_, and SO_2_F_2_ gas molecule adsorption on the anatase (001) defect surface

**Figure 8:**
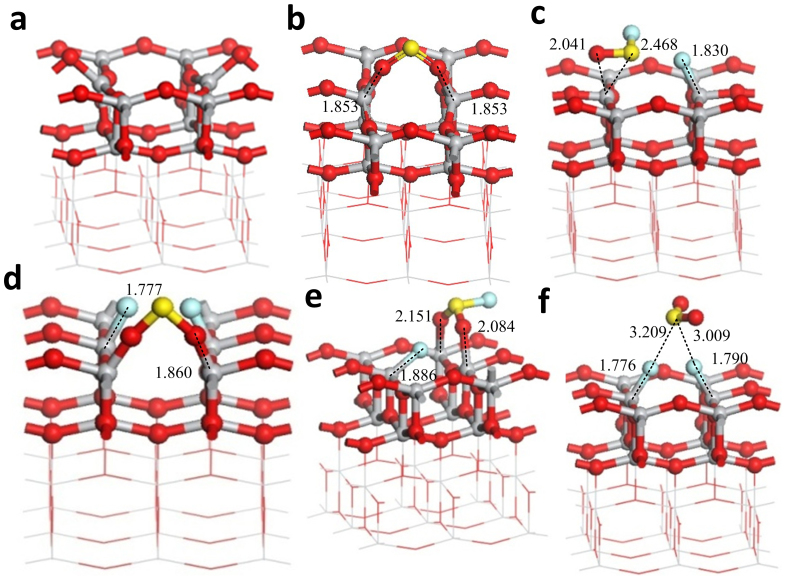
Adsorption structures of the gas molecules on the (001) defect surface. (a) The clear (001) defect surface. (b) SO_2_ adsorbs on the (001) defect surface. (c) SOF_2_ adsorbs on the (001) defect surface in the initial position O-Ti_4c_, S-Ti_4c_. (d) SOF_2_ adsorbs on the surface in F-Ti_4c_, F-Ti_4c_. (e) SO_2_F_2_ adsorbs on the surface in F-Ti_4c_, O-Ti_4c_. (f) SO_2_F_2_ adsorbs on the surface F-Ti_4c_, F-Ti_4c_. Binding distances are in Å.

Figure 8 shows the stable adsorption structure of the three gas molecules on the (001) defect surface. Comparison with the (101) defect surface reveals that the active atoms of (001) defect surface are both Ti_4c_. The interactions between the oxygen vacancy of the (001) surface and the gas molecules are stronger than those of the (101) surface based on the adsorption energy. Adsorption of SO_2_ on the oxygen vacancy results in the interaction between the two O atoms of SO_2_ and the two Ti_4c_ to form two O-Ti_4c_ bonds. Similar to the (101) defect surface, different adsorption structures exist when SOF_2_ and SO_2_F_2 _are adsorbed on the (001) defect surface.

The initial positions of SOF_2_ approach the vacancy: 1) O-Ti_4c_, S-Ti_4c_, 2) F-Ti_4c_, O-Ti_4c_, 3) F-Ti_4c_, F-Ti_4c_. The first and the second stable adsorption structures are similar (Figure 8(c)). One F atom bonds with Ti_4c_, and SOF is above the other Ti_4c_. The two S-F bonds break, and the two F atoms respectively interact with the two Ti_4c_ (Figure 8(d)). SO is adsorbed on the adjacent O_2c_ and is similar to that of SO_2_ on the oxygen vacancy.

The initial positions of SO_2_F_2_ approach the vacancy: 1) O-Ti_4c_, O-Ti_4c_, 2) F-Ti_4c_, O-Ti_4c_, 3) F-Ti_4c_, F-Ti_4c_. The adsorption energy of first structure is also small but higher than that of the (101) defect surface. The two S-F bonds do not break, and the molecular shape of SO_2_F_2_ markedly changes. Figures 8(e) and 8(f) show the latter two stable adsorption structures. The two S-F bonds break, and the two F atoms respectively interact with the two Ti_4c_ to yield SO_2_ (Figure 8(f)). One S-F bond breaks and the F atom of this bond interacts with Ti_4c_; one O atom interacts with Ti_4c_, and the other S-F bond does not break (Figure 8(e)).

**Figure 9:**
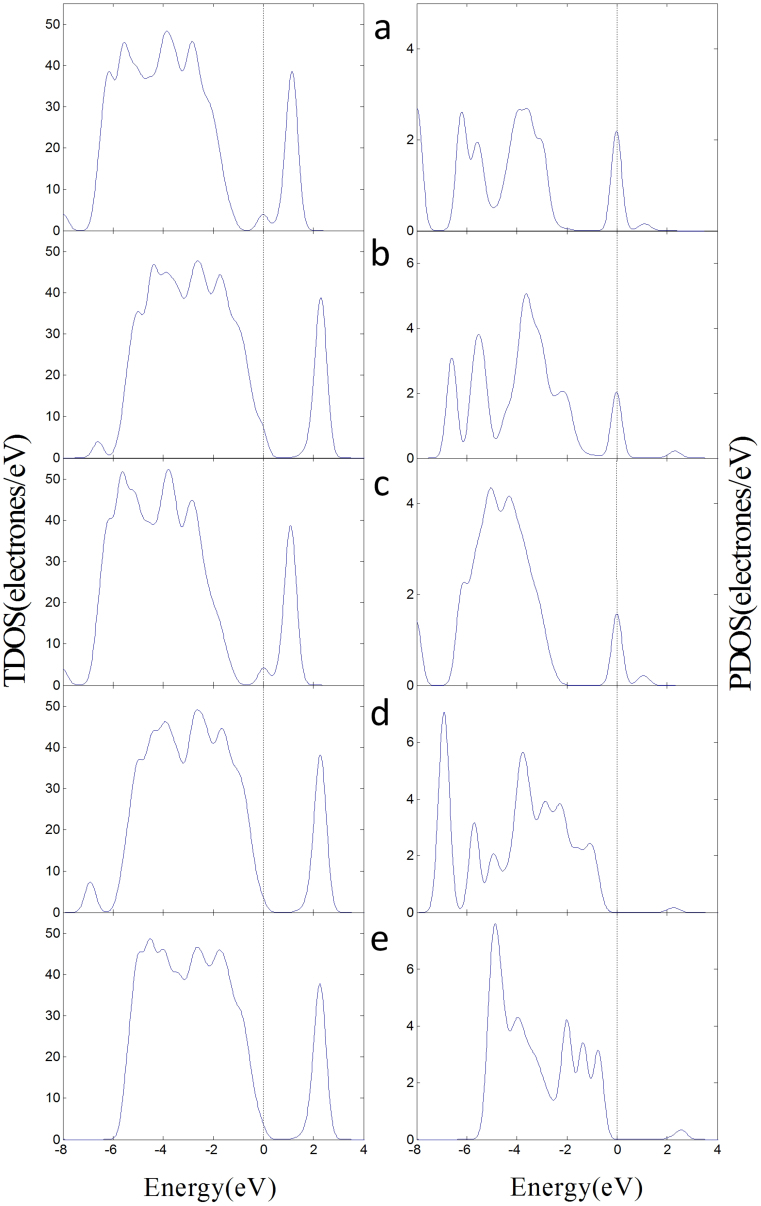
DOS of the adsorption structures on the (001) defect surface. (a) The TDOS of SO_2_ adsorbs on the (001) defect surface in left and the PDOS of SO_2_ in right. (b) SOF_2_ adsorbs on the (001) surface in the initial position O-Ti_4c_, S-Ti_4c_. (c) SOF_2_ adsorbs on the surface in F-Ti_4c_, F-Ti_4c_. (d) SO_2_F_2_ adsorbs on the surface in F-Ti_4c_, O-Ti_4c_. (e) SO_2_F_2_ adsorbs on the surface F-Ti_4c_, F-Ti_4c_.

Compared with the (101) defect surface, the oxygen vacancy of the (001) surface has a stronger adsorption interaction with the three gas molecules. The two active Ti_4c_ are easier to bond with the F atoms of SOF_2_ and SO_2_F_2_. Figure 9 illustrates the TDOS of the adsorption structures of the (001) defect surface and the PDOS of the adsorbed gas molecules. The changes in the DOS induced by the gas molecules in the (001) defect surface adsorption structures are similar to that in the (101) defect surface. Here, the adsorbed SO_2_ and SOF_2_ molecules induced the impurity state between the VB and CB of the adsorption structures and improved the conductivity of the (001) defect surface. The adsorbed SO_2_F_2_ relatively widens the band gap and reduces the conductivity of the (001) defect surface.

### Analysis of the experimental results

**Figure 10:**
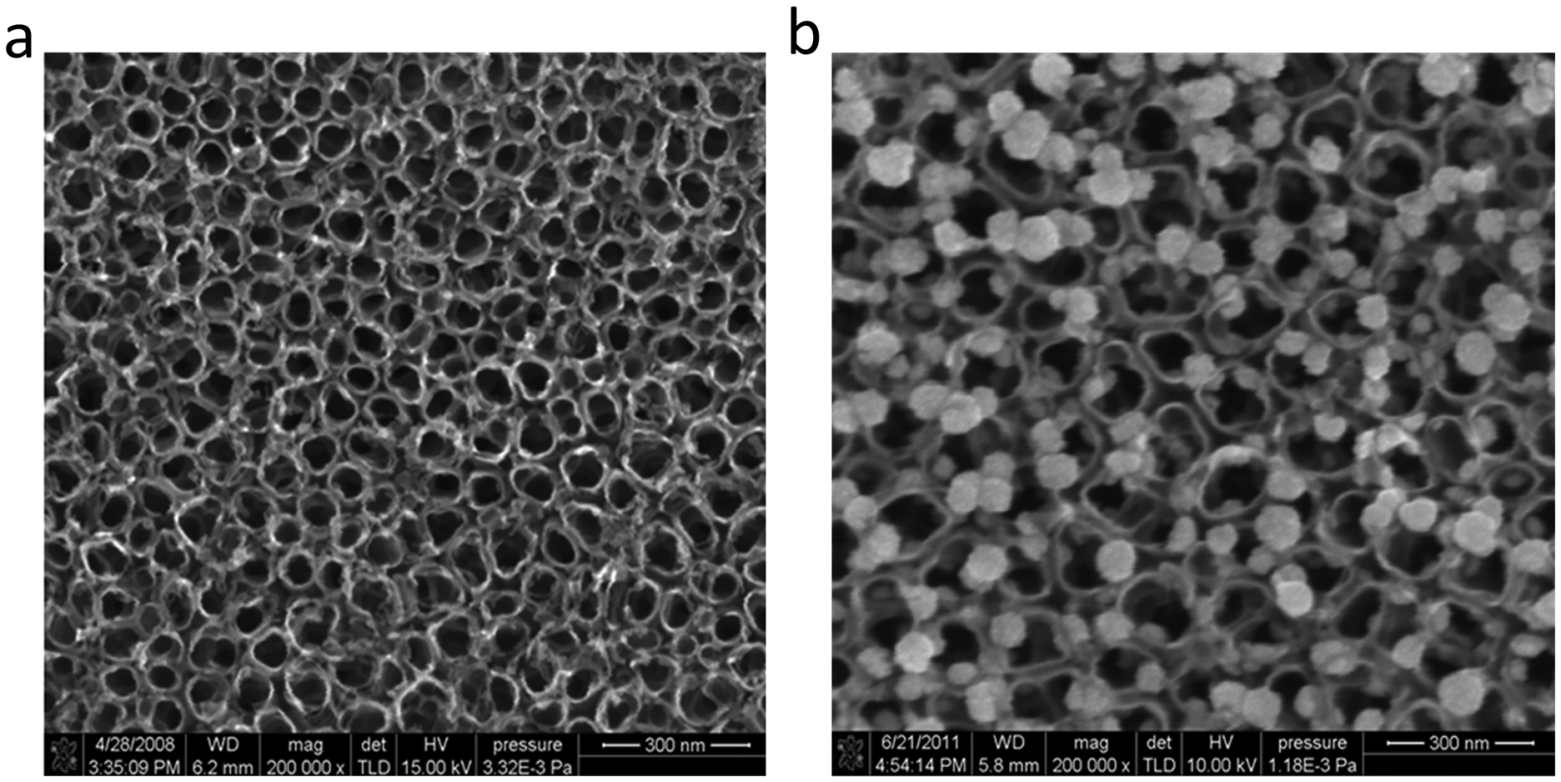
SEM images of intrinsic and Pt-doped TNTAs. (a) The intrinsic TNTAs prepared through the electrochemical anodization of titanium foil. (b) The Pt-doped TNTAs prepared by deposited Pt nanoparticles on the surface of intrinsic TNTAs.

**Figure 11:**
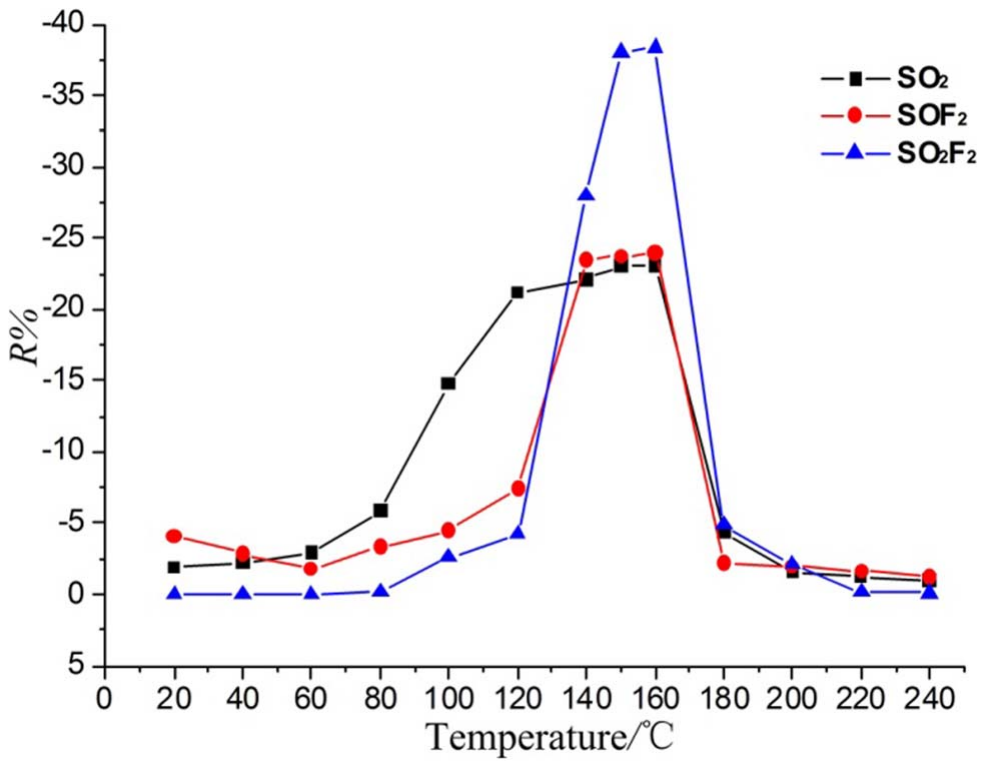
Temperature characteristic curves of the responses of Pt-doped TNTAs to 100 μL/L gases. N_2_ is used as the carrier gas in the sensing experiment. The response *R%* is defined as the relative variation of the sensor's resistance: (*R*_g_ – *R*_0_)/*R*_0_, where *R*_g_ is the resistance of the sensor to the relevant gas, and *R*_0_ is pure N_2_.

In our previous research, we analyze the sensing mechanism of the intrinsic TNTA gas sensor based on the preliminary simulation results and show that the SO_2_ adsorption introduces an impurity state at the bottom of the conductance band that narrows the band gap and enhances the electronic conductivity of the anatase (101) perfect surface^12^. Hence, the intrinsic TNTA gas sensor shows a better response to SO_2_ than SOF_2_ and SO_2_F_2_ at its working temperature. We then prepare Pt-doped TNTAs by deposited Pt nanoparticles on the surface of TNTAs. Figure 10 shows the SEMs of intrinsic TNTAs and Pt-doped TNTAs. The gas-sensing properties of Pt-doped TNTAs are test and shown in Figure 11. The Pt-doped TNTAs shows a better response to SO_2_F_2_ than SO_2_ and SOF_2_ at its working temperature, which significantly change compared with that of the intrinsic TNTA gas sensor^11^.

The Pt nanoparticles have strong catalytic performances, and the surface of Pt-deposited TNTAs is more active than the perfect surface of intrinsic TNTAs. Considering the defect surface of intrinsic TNTAs is also very active, we simulate the adsorption between the TNTA defect surface and the three gas molecules, and try to explain the sensing mechanism of Pt-doped TNTAs.

Table 1 shows that the ability to improve the conductivity of perfect surface follows the order SO_2_ > SOF_2_ > SO_2_F_2_. Table 2 and Table 3 show that the ability to improve the conductivity of defect surface nearly follows the order SOF_2_ > SO_2_ > SO_2_F_2_. It indicates that the selection of defect surface to the three gases is different from that of perfect surface. The simulation results reveal the primary difference between perfect surface and defect surface to adsorb the three gas molecules: Adsorption on the perfect surface ceases the decomposition of SOF_2_ and SO_2_F_2_, whereas that on the defect surface SOF_2_ and SO_2_F_2_ decomposes. SOF_2_ may decompose into two F atoms and one SO molecule, and SO_2_F_2_ may decompose into two F atoms and one SO_2_ molecule. SO_2_ do not decompose whether it is adsorbed on the perfect or defect surface. From here, we conclude that the selection of TNTA surface to the three gases probably depends on the decomposition of the gases in adsorptions.

**Figure 12:**
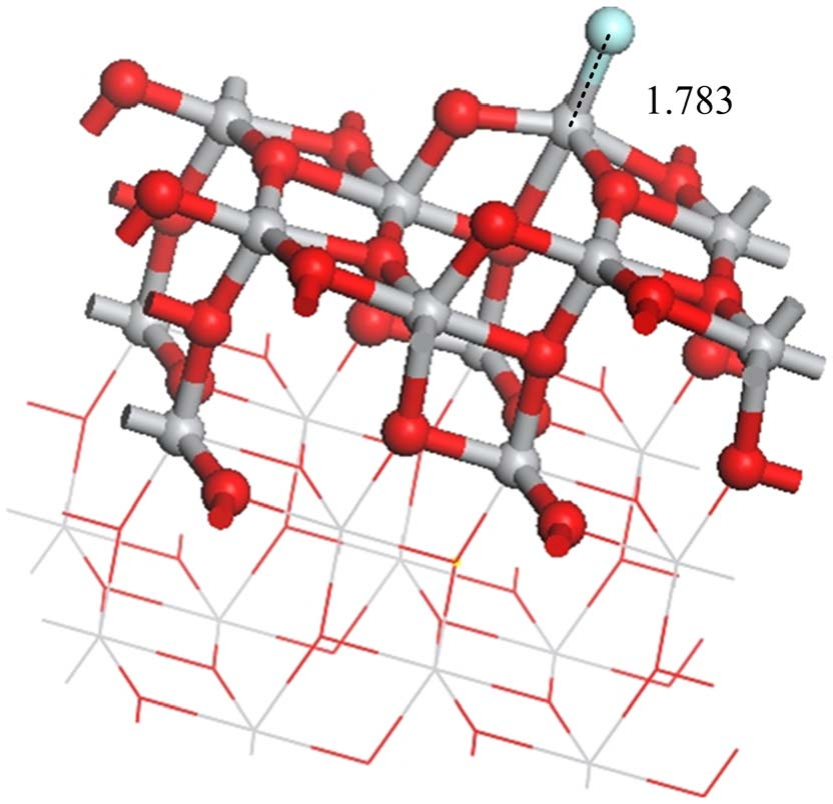
The adsorption structure of single F atom on the (101) perfect surface.

The surface of intrinsic TNTAs includes defect surface and perfect surface. The surface of Pt-doped TNTAs includes Pt-doped surface and perfect surface. SO_2_, SO and F produced in the adsorptions of SOF_2_ and SO_2_F_2_ on defect surface or Pt-doped surface may also adsorb on perfect surface. So we analyze the effects of them adsorbing on the (101) perfect surface. Figure 12 shows the single F atom adsorbs on the (101) perfect surface. The adsorbed F atom reduces the band gap of (101) perfect surface from 1.951 eV to 1.937 eV, whereas the adsorbed SO_2_ reduces the band gap of (101) perfect surface from 1.951 eV to 1.788 eV. Thus, SO_2_ molecule is better than single F atom to improve the conductivity of the (101) perfect surface. Figure 6(b) shows the SO_2_ adsorbs on the (101) defect surface and is similar to the SO adsorbs on the (101) perfect surface. When SO adsorbs on the (101) perfect surface, the band gap is 1.524 eV. This value is lower than that of SO_2_ adsorbing on the (101) perfect (1.788 eV). So the ability to improve the conductivity of (101) perfect surface follows the order SO > SO_2_ > F. It indicates that once SOF_2_ and SO_2_F_2_ decompose in adsorptions, the effects of them to the conductivity of the TNTA surface change.

The surface of intrinsic TNTAs has few oxygen defects and is mostly the perfect surface. The perfect surface is more sensitive and selective to SO_2_ than to SOF_2_ and SO_2_F_2_, so the gas responses of intrinsic TNTAs appear as the curves in the Ref. 10. Compared to the surface of intrinsic TNTAs, the surface of Pt-doped TNTAs may have more active catalytic centers^23^. Similar to the adsorption on active oxygen defects, SOF_2_ and SO_2_F_2_ may also decompose in the adsorption upon the active centers of Pt-doped surface, which needs further investigation, thus Pt-doped TNTAs have new response curves (Figure 11). Base on this conclusion, we analyze the gas-sensing response curves of Pt-doped TNTAs below:

When the temperature is below 80°C, the responses of three gases are all small. The SO_2_F_2_ has almost no response, while the responses of SO_2_ and SOF_2_ are greater than that of SO_2_F_2_ because SO_2_F_2_ has the lowest HOMO and highest LUMO in three gases.

When the temperature is between 80 and 120°C, the responses of Pt-doped TNTAs and intrinsic TNTAs to the three gases are the same: SO_2_ > SOF_2_ > SO_2_F_2_. Under this condition, the response is still decided by the perfect surface of Pt-doped TNTAs but no the Pt-doped surface, and the Pt nanoparticles of Pt-doped surface are not active at this temperature.

When the temperature is between 120 and 170°C, the responses of Pt-doped TNTAs to the three gases follow the order SO_2_F_2_ > SOF_2_ > SO_2_, which markedly change, while that of intrinsic TNTAs still follow the order SO_2_ > SOF_2_ > SO_2_F_2_. Under this temperature, the Pt nanoparticles on TNTAs surface become active. The adsorbed SOF_2_ and SO_2_F_2_ may decompose in the adsorptions upon the active centers of Pt-doped surface, leading to the changes in responses.

When the temperature is >170°C, the responses of the three gases are very low, because it is not easy for the gas molecules to adsorb on TNTAs surface under high temperature, and the adsorption quantity decreases. The Pt-doped TNTAs have a good conductivity at high temperatures, which is insensitive to adsorbed gas molecules. Thus, the responses are low.

## Conclusions

In this work, we performed DFT calculations to study the structural and electronic properties of the anatase surfaces and their adsorptions of SF_6_ decomposed gases, namely, SO_2_, SOF_2_, and SO_2_F_2_. The calculation results were analyzed to explain the results of the Pt-doped TNTA gas sensor sensing experiment.

The main conclusions of this study are as follows:

1. The anatase (001) perfect surface has a stronger interaction with the three gases than that of anatase (101) perfect surface, and both surfaces are more sensitive and selective to SO_2_ than to SOF_2_ and SO_2_F_2_.
2. The anatase defect surface has a stronger interaction with the three gases than the perfect surface, because the oxygen vacancy on defect surface is very active. When SOF_2_ and SO_2_F_2_ adsorb on the oxygen vacancy of defect surface, these compounds may decompose in adsorption process because their S-F bonds are easier to break compared with the adsorption on the perfect surface. The by-products SO and SO_2_ will also adsorb on the surface.
3. The surface of intrinsic TNTAs has few active oxygen defects, and the deposited Pt nanoparticles increase active centers of the surface. The active centers of Pt-doped surface probably are similar to the active oxygen defects, thus the Pt-doped TNTAs have different responses to the three gases compared with the intrinsic TNTAs.

The work is expected to improve our insight into the interactions between the gases and the TNTA surface for better sensor design.

## Author Contributions

X.X.Z. designed the project, supervised the experiments and modified the manuscript. Q.C.C. performed the simulation calulations and wrote the manuscirpt. J.T. modified the manuscirpt. W.H.H. supervised the experiments and modified the manuscript. J.B.Z. did sensing experiments on gas and analyzed the data.

## Figures and Tables

**Table 1:** Calculated adsorption energy, charge transfer, and binding distance of the perfect surfaces

Surface	(101) perfect surface				(001) perfect surface			
Structure	TiO_2_(101)	SO_2_	SOF_2_	SO_2_F_2_	TiO_2_(001)	SO_2_	SOF_2_	SO_2_F_2_
*E_a_*(eV)	**\**	−0.360	−0.297	−0.214	**\**	−1.660	−1.170	−0.690
Q(e)	**\**	0.097	0.051	0.010	**\**	−0.356	−0.118	0.014
d(Å)	**\**	2.457	2.490	3.198	**\**	1.724	2.075	2.961
*E_g_*(eV)	1.951	1.788	1.932	1.936	1.613	1.488	1.565	1.602

**Table 2:** Calculated adsorption energy, charge transfer, and binding distance of the (101) defect surface

Surface	(101) defect surface					
Structure	TiO_2_(101)	SO_2_ (b)	SOF_2_ (c)	SOF_2_ (d)	SO_2_F_2_ (e)	SO_2_F_2_ (f)
*E_a_* (eV)	\	−2.150	−2.095	−3.037	−4.356	−4.686
Q(e)	\	−0.699	−0.183	−0.734	−0.978	−0.877
d(Å)	\	1.976	1.790	1.670	1.869	1.794
*E_g_* (eV)	1.888	1.524	1.250	1.283	1.932	1.935

**Table 3:** Calculated adsorption energy, charge transfer, and binding distance of the (001) defect surface

Surface	(001) defect surface					
Structure	TiO_2_(001)	SO_2_ (b)	SOF_2_ (c)	SOF_2_ (d)	SO_2_F_2_ (e)	SO_2_F_2_ (f)
*E_a_*(eV)	\	−3.205	−3.095	−4.810	−4.807	−4.786
Q(e)	\	−0.603	−0.701	−0.996	−0.978	−0.929
d(Å)	\	1.925	1.830	1.777	1.886	1.776
*E*_g_(eV)	1.385	1.263	1.469	1.204	1.562	1.499
